# Molecular Imaging in Pediatric Brain Tumors

**DOI:** 10.3390/cancers11121853

**Published:** 2019-11-23

**Authors:** Agostino Chiaravalloti, Luca Filippi, Maria Ricci, Andrea Cimini, Orazio Schillaci

**Affiliations:** 1 Department of Biomedicine and Prevention, University Tor Vergata, 00133 Rome, Italyorazio.schillaci@uniroma2.it (O.S.); 2 Nuclear Medicine Section, IRCCS Neuromed, 86077 Pozzilli, Italy; 3 Nuclear Medicine Section, “Santa Maria Goretti” Hospital, 04100 Latina, Italy; lucfil@hotmail.com; 4 Department of Radiological, Oncological and Pathological Sciences, Faculty of Medicine and Surgery, La Sapienza University, 00161 Rome, Italy; maria.ricci28@gmail.com

**Keywords:** pediatric brain tumors, molecular imaging, nuclear medicine, positron emission tomography, single-photon emission computed tomography, radiopharmaceuticals, PET, SPECT

## Abstract

In the last decade, several radiopharmaceuticals have been developed and investigated for imaging in vivo of pediatric brain tumors with the aim of exploring peculiar metabolic processes as glucose consumption, amino-acid metabolism, and protein synthesis with nuclear medicine techniques. Although the clinical shreds of evidence are limited, preliminary results are encouraging. In this review, we performed web-based and desktop research summarizing the most relevant findings of the literature published to date on this topic. Particular attention was given to the wide spectrum of nuclear medicine advances and trends in pediatric neurooncology and neurosurgery. Furthermore, the role of somatostatin receptor imaging through single-photon emission computed tomography (SPECT) and positron emission tomography (PET) probes, with reference to their potential therapeutic implications, was examined in the peculiar context. Preliminary results show that functional imaging in pediatric brain tumors might lead to significant improvements in terms of diagnostic accuracy and it could be of help in the management of the disease.

## 1. Introduction

Pediatric brain tumors (PBT) include different tumor entities of varying malignancy. The incidence rate of childhood and adolescent primary malignant and nonmalignant brain and other central nervous system tumors in the United States is approximately 5.67 per 100,000 person-years [1]. In Europe, a positive trend in incidence has been sought until the end of the ’90s (on average by 1.7% per year) [2], and an annual incidence rate of 6.8 per million children (0–14 years) was reported from Europe for the period 2000–2007 with 15–20% of all CNS tumors being Medulloblastomas, according to www.RARECAREnet.eu. PBT shows a strong histological heterogeneity [3], with the tumor location being, in many cases, proximity to critical or eloquent brain structures precludes the complete and partial resection and allows for diagnostic (stereotactic) biopsy only [3]. Magnetic Resonance Imaging (MRI) is currently the standard imaging technique for brain tumors due to its capabilities in detecting enhancement characteristics, necrosis, and the extent of the enhancing portion of the tumor in pre- and post-contrast T1 sequences and peri-tumoral edema (vasogenic and infiltrative), blood products, calcifications, radiation-induced chronic micro-hemorrhages, and non-enhancing tumor in T2 sequences [4,5]. Moreover, peculiar sequences allow for the detection, among others, of tumor vascularity and tractography for surgical planning or navigation [5]. In the diagnosis of PBT, MRI could show several non-specific findings, such as hyperintensity on T2-weighted images and fluid-attenuated inversion recovery (FLAIR), which might limit the diagnostic accuracy [6,7]. These limitations are relevant especially when surgery is associated with adjuvant therapy. This might induce tissue changes that need to be differentiated from residual viable tumor tissue or tumor progression. The challenge is mostly represented by the differentiation of treatment effects, as radiation-induced changes, from the recurrent or progressive disease. Several efforts have been undertaken for the creation of standardized MRI-based criteria for response assessment in neurooncology (RANO) that are applied today in patients with brain tumors [8]. Nevertheless, contrast enhancement as detectable in MRI contrast enhancement is a sub-optimal biomarker for the detection of tumor size or growth [8]. Contrast enhancement, in fact, reflects both the vascular surface area and the permeability of the contrast agent across a disrupted blood-tumor barrier [8]. There are several factors that are related to tumor biology therapy-induced inflammation that can affect blood-tumor barrier permeability. Moreover, therapeutics that affect tumor vascular permeability, such as corticosteroid, antiangiogenic, or immunotherapy agents, can influence contrast enhancement [9]. Therefore, additional diagnostic techniques that are capable of investigating different metabolic processes are helpful in the characterization of PBT at diagnosis and in follow-up after treatment [6,7].

Molecular imaging (MI) provides insight into pathological processes at a cellular and molecular level. If conventional imaging as computed tomography (CT) and MRI predominantly offer anatomical details of the disease, molecular imaging allows for the detection of those biological processes that often precede morphological changes of the disease. There are several positive outcomes of MI in the clinical settings that enable physicians to personalize patient care. For diagnostic purposes, MI provides information that is achievable with more invasive procedures as biopsy or surgical excision and allows for the detection of the disease at its earliest stages and its exact location. In the management of the care of the patients, MI plays a relevant role in determining the extent or severity of the disease (by a correct staging with accurate detection of distant metastases) and the selection of the most appropriate therapy for the molecular properties of the disease. Several reports cited in the following chapters demonstrate that MI is capable of accurately determining the patient’s response to therapy, thus modulating the therapy approach on the basis of the response. Moreover, the early detection of recurrence helps the management of ongoing care.

Positron emission tomography/Computed tomography (PET/CT) and single-photon emission computed tomography (SPECT) represent two diagnostic imaging modalities used in nuclear medicine for the evaluation of the bio-distribution of "radioactive" preparations (radiopharmaceuticals) in different clinical settings; radiopharmaceuticals own chemical-physical-biological characteristics that comply with all of the regulations of the official pharmacopeia for administration in humans. Today, numerous radiopharmaceuticals are available that can electively concentrate in different tissues and organs, thus allowing for the study of their morpho-functional characteristics.

In a recent review of Fei B et al., the biopsy guided by MI with PET showed promising results in several reports [10]. For the brain, PET was superior for neurosurgical biopsy guidance, resection planning, and radiotherapeutic target volume delineation in several studies as compared to MRI [10]. Information provided by MI leads to incremental benefit during the performance of image-guided biopsies in bone, lung, lymph nodes, and soft-tissue masses. PET/CT data correctly guided the needle placement in the viable portion of a lesion, which increased the chances of achieving an accurate diagnosis [10]. The feasibility and potential value of MI were investigated to improve the accuracy of targeted lymph node biopsy during mediastinoscopy [10]. MI plays a central role in the management of radiation oncology patients [11]. Before radiation therapy, the crucial role of MI in diagnosis and staging allows for better target definition. In the last decade, several technical advances allow for precise and conformal delivery of dose to patients, but a correct volume delineation that includes the greatest part of the viable tumor is crucial. MI has proved to be useful in reducing the interobserver variability in target volume delineation and in bringing about greater conformity between the target volume boundaries and true tumor boundaries [11]. During the treatment and after radiotherapy, MI allows for a more complete treatment response assessment [11]. MI is an early predictor of treatment outcome and it allows therapy management to optimize the treatment. After the treatment, MI serves as a predictor of therapy response. Anatomic changes that are visible on CT or MRI occur late after response and they may require weeks or months, whereas the molecular imaging treatment response can be potentially assessed much earlier [11].

The aim of this review is to provide a comprehensive overview of the usefulness and limitations of nuclear medicine in the evaluation of PBT. Table 1 provides a general overview of the studies cited in this review (that includes a short summary of the most relevant findings), while Table 2 summarizes the advantages and drawbacks of the different tracers included in the manuscript.

## 2. Imaging of Cell Metabolism (Buildup of Phospholipid Cell Membranes [^11^C] Choline ([^11^C] CH), [^18^F] Fluoromethylcholine ([^18^F] FCH) and [^18^F] Fluoroethylcholine ([^18^F] FEC)

Phosphatidylcholine represents an essential component of cellular membranes and its synthesis starts from choline substrate through the CDP-choline pathway. Malignant cells are characterized by high levels of choline-kinase activity, which in turn entails an increased utilization of choline, due to their increased rate of proliferation. Choline (*methyl*-choline) was firstly labeled with the radionuclide [^11^C] carbon and successfully applied for the imaging of tumors, especially prostate cancer, through positron emission tomography (PET) [51]. After its administration, radiolabeled choline is rapidly incorporated into malignant cells, phosphorylated to become phosphorylcholine, and then finally integrated into cell membranes as phosphatidylcholine. The most relevant drawback of [^11^C]-labeling is the short half-life of the radioisotope (about 20 min), therefore the clinical use of this tracer is feasible only in centers equipped with an on-site cyclotron. Two choline derivatives labeled with [^18^F] fluorine, which has a significantly longer half-life (i.e., 110 min), have been introduced in clinical practice to overcome this limitation: ^18^F-*methyl-*choline ([^18^F] FCH) and ^18^F-*ethyl-*choline, ([^18^F) FEC) [52,53]. The former compound was introduced by DeGrado and coworkers [54], who demonstrated that [^18^F] FCH strictly follows the same mechanisms by which choline is incorporated and metabolized into tumoral cells since the in vitro uptake of the [^18^F]-labeled tracer resulted in being significantly reduced by a specific choline-inhibitor. Of note, as regards brain tumors, the profile of uptake for both [^18^F] FCH and [^11^C] choline was investigated in glioma cell culture: the authors found that the metabolism of (^18^F) FCH was very close to that of choline, although some minor differences were registered between the two compounds [55]. As concerns the latter [^18^F]-derivative compound (i.e., [^18^F] FEC), in first preliminary reports from Hara et al., the tracer was found to have a very similar biodistribution to that of the [^11^C]-labeled choline with the only remarkable difference being represented by its more rapid urinary excretion [56]. Of note, [^18^F] FEC showed similar uptake characteristics as [^11^C]choline, both in culture tumoral cells and in the first studies that were performed in patients affected by prostate cancer.

As specifically regarding the imaging of brain tumors, George et al. [57] proved that [^3^H] labeled choline is incorporated into cultured glioma cells through a specific transport system. The first studies in humans demonstrated that [^11^C] choline presents a very rapid blood clearance, with stable distribution in tissues and organs after the first 5 min p.i. The normal biodistribution in the brain includes very low uptake in normal parenchyma, relatively intense physiological retention in the choroid plexus of the left ventricles, and in the nasal mucosa. In a report by Hara et al., PET with [^11^C] choline was found to be able to clearly identify brain tumors in 31 adult patients [58]. Of note, in the cited paper, all of the patients underwent a cerebral blood flow measurement by PET with i.v. administration of [^15^O] water before the PET study with [^11^C] choline. The authors did not find any meaningful correlation between the grade of [^11^C] choline uptake and the entity of blood flow. Furthermore, it has to be pointed out that the avidity of brain tumors for radiolabeled choline is in substantial agreement with a high content of choline-containing compounds that was revealed in these malignancies by MRI spectroscopy, while low or undetectable choline content is present in normal brain tissue [59]. 

As regards pediatric brain tumors, Ohtani and colleagues reported a first experience [15], who prospectively evaluated the usefulness of PET/CT with [^15^O] choline in 22 untreated patients with brain tumors, including three children affected, respectively, by pilocytic astrocytoma (pons), glioblastoma (pons), and dysplastic glioneuronal lesion (cerebellum). In such patients, [^11^C] choline, PET with 2-deoxy-2-[^18^F] fluoro-D-glucose ([^18^F] FDG, half-life 108 min) and MRI were performed within two weeks of each other. Afterward, every subject underwent neurosurgery and histopathological examination. PET with [^11^C] choline performed better than that with [^18^F] FDG for the detection of cerebral malignancies and it was able to differentiate between the high-grade and low-grade gliomas, but it failed in discriminating low-grade gliomas from non-neoplastic lesions (i.e., the dysplastic glioneuronal lesion). In light of the foregoing, the authors suggested the combined use of PET with [^11^C] choline and MRI as a useful imaging approach for the characterization of brain lesions. Section 4 describes the characteristics and usefulness of [^18^F] FDG in more detail.

**Figure 1:**
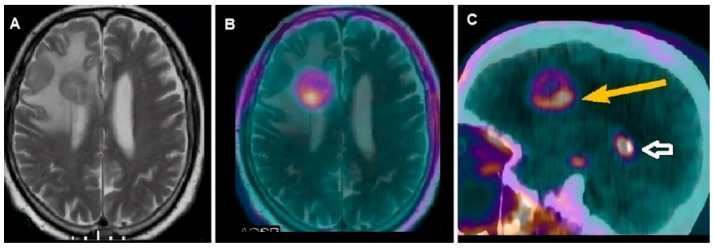
16-year-old male patient affected by astrocytoma, treated with radiotherapy and suspected for relapse at Magnetic Resonance Imaging (MRI) examination (**A**). Off-line co-registered [^18^F] choline PET/MRI (**B**) axial slice well demonstrated intense tracer in the peripheral and posterior part of the large morphological lesion detected by MRI, thus confirming the diagnosis of recurrent tumor. In (**C**), sagittal fused PET/CT slice well-depicted tracer uptake in the tumor (yellow arrow), also showing the physiological uptake in the choroid plexus (white bordered arrow).

As concerns [^18^F] radiolabeled choline derivatives, a paper by Fraioli et al. [16] investigated the role of PET/MRI scan with [^18^F] FEC in 12 children bearing brain tumors, eight of whom were also evaluated with a follow-up PET/MRI at three and six months after the completion of chemotherapy and radiotherapy. The authors evaluated the feasibility of the examination by using a hybrid PET/MRI scanner and also assessed the correlation between PET-derived parameters (i.e., SUV_max-mean_) and apparent diffusion coefficient (ADC) maps that were obtained through MRI. All of the patients were affected by astrocytic tumors: at baseline, an agreement between the areas of ^18^F-cho uptake and the enhancement pattern revealed by MRI was found. Image analysis revealed no meaningful correlation between SUV_max_ and ADC_mean_ values, SUV_max_ and size, or ADC_mean_ and size. However, a negative correlation trend was pointed out between SUV_max_ and ADC_mean_ and a positive correlation trend was registered between SUV_max_ and tumor size. As concerns the eight patients examined after therapy, four of them presented lesions slightly decreased in size at the first follow-up. In such cases, the authors registered a decrease in SUV_max_ (in four cases) and an increase in ADC_mean_ (three cases), as compared to the values that were observed at baseline. The same authors suggest that this inverse correlation in responder-patients might be explained by the reduced tumor cellularity that, on one side, leads to a reduced restriction of free water movement (increased ADC values) and on the other it entails a reduced consumption of radiolabeled choline (decreased SUV_max_). In conclusion, the integrated utilization of PET and MRI technology through the hybrid device, even still to be validated in larger series, was suggested as a powerful diagnostic tool for the staging and restaging of pediatric astrocytomas (Figure 1).

Tsouana et al. [17] investigated the role of hybrid PET/MRI with [^18^F] FEC for the diagnosis, assessment of treatment response, and remission status in a case series of four adolescent patients with suspected (*n* = 1) or proven (*n* = 3) non-germinomatous germ cell tumors (NGGCT). In one patient, baseline PET-MRI detected a pineal cystic lesion with no [^18^F] FEC uptake. In this subject with negative morpho-functional findings, an open biopsy was performed and histology was negative for tumors due to the extremely increased beta-HCG level. The other three patients with the mass in the pineal region, elevated values of AFP and beta-HCG, showed intense [^18^F] FEC uptake in the cerebral lesions at baseline PET/MRI. In such cases, the diagnosis of NGGCT was made and chemotherapy was commenced. [^18^F] FEC PET/MRI was repeated to assess the response to therapy: a significant reduction in volume and uptake was observed in all of the examined subjects. PET-MRI findings were found to be consistent with the subsequently performed surgery and histology.

## 3. Imaging of Somatostatin Receptors: (^111^Indium-Diethylenetriaminepentaacetic Acid-d-Phenylalanine-Octreotide ([^111^In] Pentetreotide), [^68^Ga] DOTA-Peptides)

Somatostatin is a peptide hormone that is produced by the hypothalamus. It regulates a wide variety of physiological functions and presents an inhibitory effect on the secretion of other hormones, on the motility of the gastrointestinal tract and cell proliferation [60]. The function of somatostatin is mediated by specific receptors (*sstr*) that belong to the 7-transmembrane G protein-coupled receptor superfamily that is physiologically expressed in the central and peripheral nervous system, in the endocrine portion of the pancreas and in the gastrointestinal tract. Five different subtypes of *sstr* were identified (i.e., *sstr*1-5). Tumors of neuroendocrine origin (NET), which arise from the neuroendocrine cells in the lung or in the bowel, were found to overexpress *sstr* on their cell membranes, in particular, the subtype 2 (*sstr*2). As specifically regarding pediatric tumors, *sstr*2 were disclosed in embryonal malignancies, such as neuroblastoma, medulloblastoma, and supratentorial primitive neuroectodermal tumor (PNET) [61].

Native somatostatin peptide is rapidly inactivated by the blood enzymes and it presents a half-life of <2 min., thus its application as an imaging agent is unfeasible. A synthetic chelated analog of somatostatin pentetreotide, labeled with the radionuclide [^111^In] with a physical half-life of 67.2 h, proved to present high affinity for sstr2 and lower affinity to sstr3 and sstr5, while not significant binding was reported for sstr1 and sstr4 [62]. [^111^In] pentetreotide has been introduced in the clinical practice for the imaging of NETs through the conventional scintigraphic (planar and SPECT) technique. The scintigraphy of somatostatin receptors was extensively used over the past 20 years for a NET diagnosis, prognosis, and evaluation after therapy, but its sensitivity is strictly limited by the dimension of the lesion [63]. Three radiopharmaceuticals have been recently developed for the imaging of NET through PET technology, which is characterized by higher spatial resolution, it is a single-day procedure, and can provide semiquantitative measurements, such as the SUVmax-mean, to overcome these limitations. The three compounds, labeled with the radionuclide gallium-68 ([^68^Ga]) and commonly used for PET imaging in clnical practice, are: [^68^Ga] DOTAPhe1-Tyr3-Octreotide (DOTATOC), [^68^Ga] DOTA-NaI3-Octreotide (DOTANOC), and [^68^Ga] DOTA-Tyr3-octreotate (DOTATATE) [64,65]. The radionuclide [^68^Ga] presents a half-life of 67.71 min, is easily available through the [^68^Ge]/[^68^Ga] generator, and can be applied in radiopharmaceutical preparations by using the bifunctional chelator (BFC) acting as a bridge between [^68^Ga] and several biomolecules [66]. The most common BFC is represented by DOTA (1,4,7,10-tetraazacyclododecane-1,4,7,10-tetraacetic-acid)m which has been widely used for conjugating [^68^Ga] with the three different peptides [i.e., -TOC, -NOC, -TATE) binding to *sstr*. Although they are characterized by minimal differences in affinity for *sstr*, the three aforementioned [^68^Ga] DOTA-peptides have been demonstrated to be highly sensitive and specific in detecting well differentiated NET, with a higher detection rate when compared to conventional radiological imaging (i.e., CT/MRI) [67]. As concerns the applications in pediatric imaging, promising results were reported for the use of [^68^Ga] DOTATATE for the imaging of neuroblastoma, especially in the case of relapsed tumors [68]. In the following, we will review the application of somatostatin receptor imaging (SRI) with [^111^In] pentetreotide or [^68^Ga] DOTA-peptides for the diagnosis and follow-up of PBT.

**Figure 2:**
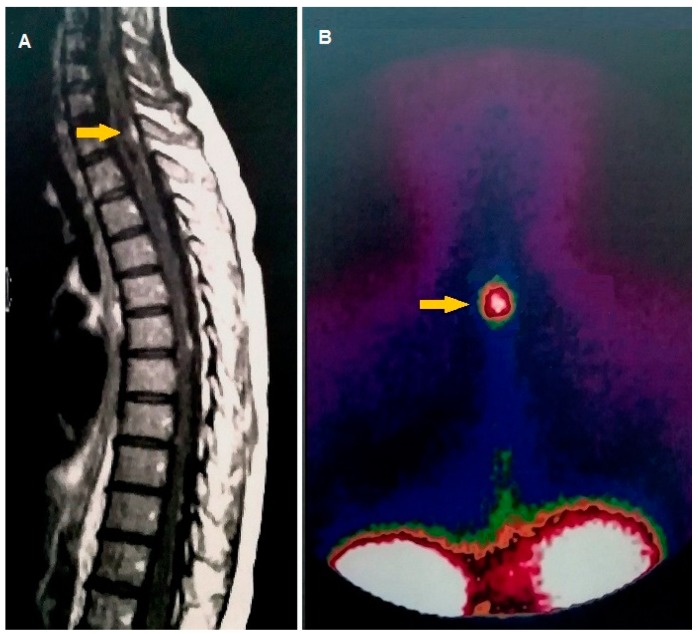
12-year-old female previously treated with surgery and chemotherapy for medulloblastoma of the posterior fossa. Sagittal contrast T1-weighted MRI shows nodular metastases of the spinal cord. The largest metastasis depicted by MRI (**A**, yellow arrow) was characterized by increased incorporation of [^111^In] pentetreotide at somatostatin receptor scintigraphy, as shown by posteroanterior planar projection (**B**, yellow arrow). The cerebrospinal fluid examination confirmed the diagnosis of leptomeningeal metastasis.

**Figure 3:**
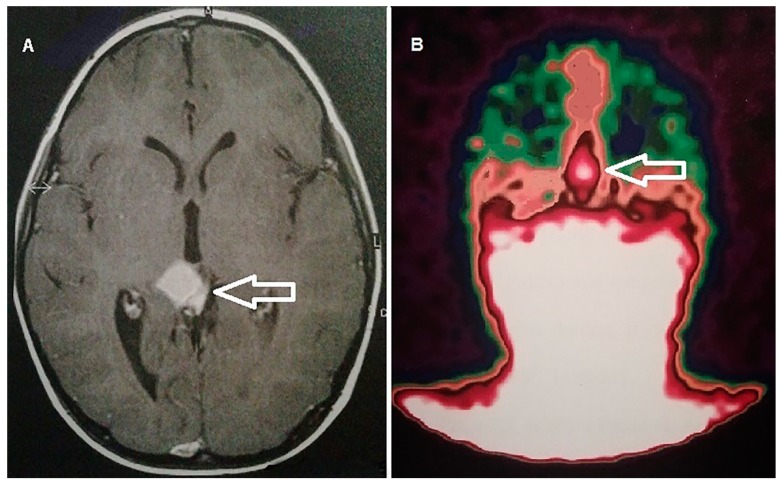
An eight year-old-male patient with sudden onset of nausea/vomiting, headache, and diplopia. An MRI scan (**A**) disclosed a large tumor (white bordered arrow) in the pineal region of the brain. Somatostatin receptor scintigraphy with [^111^In] pentetreotide (**B**) demonstrated intense tracer uptake corresponding to the brain lesion (white bordered arrow), consistent with high somatostatin receptor (subtype 2) expression. The patient underwent surgery. The final diagnosis was pineoblastoma.

As early as in 1998, Muller et al. evaluated the potential usefulness of SRI in children that were affected by medulloblastoma, one of the most common pediatric solid tumors [18]. The authors analyzed 20 patients, aged from one to 15 years old: 13 presented newly diagnosed lesions, which underwent surgical resection, while the remaining seven children presented residual tumor after therapy or CT/MRI signs of relapse. Sixteen patients were submitted to SRI with [^111^In] pentetreotide: seven patients showed high uptake in lesions, two of whom before surgery, two after subtotal resection, and the remaining three at relapse. Of note, fourteen tissue samples were obtained after surgery and then analyzed for detecting the expression of *sstr* through autoradiography: in such cases, the uptake of [^111^In] pentetreotide was consistent with the autoradiographic finding of overexpression of *sstr*2. Figure 2 shows a case of relapsed medulloblastoma with spinal metastases, while Figure 3 depicts a rare case of pineoblastoma.

An interesting report from Fruhwald and collaborators evaluated the expression of *sstr*2 in sample tissues that were obtained from pediatric patients affected by malignant central nervous system tumors [19]. The authors examined 13 patients through SRI with [^111^In] pentetreotide in a clinical setting suspected for residual tumor or relapse. In such cases, SRI resulted positive in 6/7 supratentorial PNET, in one rhabdomyosarcoma and one medulloblastoma, but neither the ependymoma nor the glioblastoma multiforme presented significant [^111^In] pentetreotide uptake in spite of severe clinical progression.

[^68^Ga] DOTA-peptides present several advantages for SRI, when compared to [^111^In] pentetreotide. First of all, the affinity of these tracers for *sstr*2 is stronger than that of [^111^In] pentetreotide and the hybrid PET/CT technology presents higher spatial resolution when compared to conventional scintigraphy (both planar and SPECT). Furthermore, the dosimetry for [^68^Ga] DOTA-compounds is also more favorable with an adult dose of 4.3 milliSieverts for 185 Megabequerels of [^68^Ga] DOTATOC versus 17.7 mSv for a typical 222 MBq administration of [^111^In] pentetreotide [69]. Nevertheless, the scientific data regarding the application of PET/CT with [^68^Ga] DOTA-peptides for the imaging of PBT is still scant.

Abongwa and collaborators enrolled two pediatric brain tumors in their study (one medulloblastoma and one supratentorial PNET) in a recently published paper focusing on the role of PET/CT with [^68^Ga] DOTATOC in solid tumors of children and young adults. In the overall examined population, which included histotypes of others than cerebral malignancies, the sensitivity and specificity of [^68^Ga] DOTATOC PET resulted in 88% and 100%, respectively [20].

Arunraj et al. documented a case of cerebellar desmoplastic medulloblastoma, which presented recurrence more than once and resulted in being positive at the examination with [^68^Ga] DOTANOC PET/CT [21]. Of note, the demonstration of affinity for [^68^Ga] DOTA-compounds in some pediatric tumors opens the door to possible therapeutic applications, since these radiopharmaceuticals can be labeled with beta-emitter radionuclides, such as ^177^Lutetium ([^177^Lu], half life: 6.647 days) or ^90^Yttrium ([^90^Y], half life: half life: 64.1 h). In this regard, it has to be mentioned the clinical phase I study performed by Menda et al. [22], who aimed to assess the safety and efficacy of a possible peptide receptor radionuclide therapy (PRRT) with [^90^Y] DOTATOC in children and young adults with refractory solid tumors expressing *sstr*2. The authors included 17 patients with at least one lesion positively imaged with [^111^In] pentetreotide. Among the enrolled subjects, three pediatric brain tumors (one astrocytoma, one pineoblastoma, and one medulloblastoma) were present. In the cited paper, PRRT was found to have a favorable safety profile with an overall response rate of 76% and kidney radiation exposure representing the main dose-limiting factor. In particular, one eleven-year-old patient with therapy-refractory anaplastic astrocytoma was reported to have a partial response, with a significant reduction in tumor size and a good impact in a child’s everyday life and activity [22].

## 4. Imaging of Cell Metabolism (Glucose Metabolism and Mitochondrial Oxidative Metabolism): ^18^F-Fluorodeoxyglucose ([^18^F] FDG) and ^99m^Tc-Methoxyisobutylisonitrile ([^99m^Tc] MIBI)

**Figure 4:**
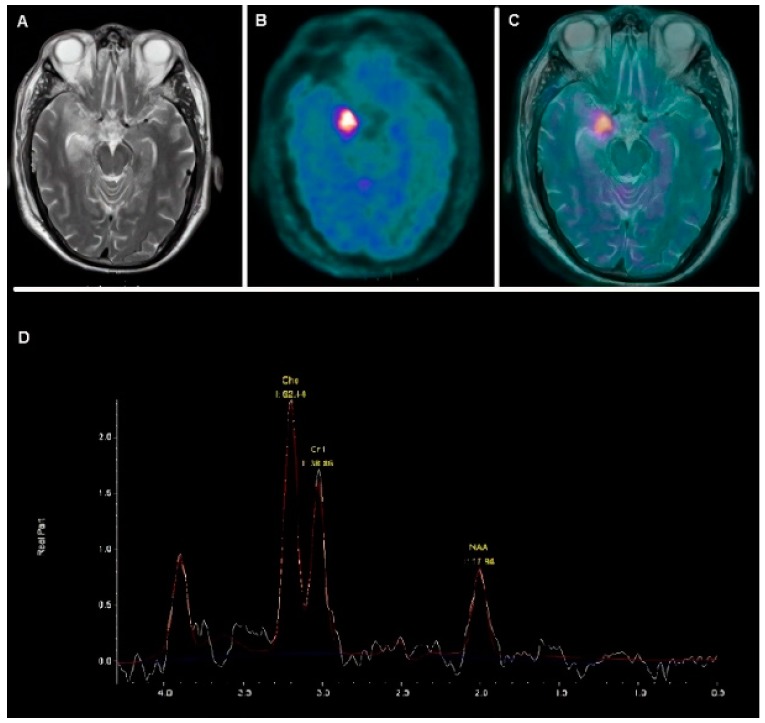
A 17-year-old male with sudden onset of difficulty speaking and understanding language, and increased aggressive behavior. MRI (**A**) demonstrated signal alteration in the right temporal lobe, in correspondence of the hippocampal/parahippocampal cortex. [^18^F] FDG PET/CT (**B**) axial slice demonstrated highly increased tracer uptake corresponding to the brain lesion, as evident from the *off-line* co-registered PET/MRI image (**C**). MR spectroscopy in (**D**) depicted the peaks of, respectively, choline (Cho), creatinine (Cr), and n-acetyl aspartate (NAA). Functional imaging and spectroscopy were consistent with the high-grade glial lesion. The patient underwent surgery. The final diagnosis was anaplastic astrocytoma.

The radipharmaceutical [^18^F] FDG is a positron-emitting radiopharmaceutical that contains no-carrier added radioactive 2-deoxy-2-[^18^F]fluoro-D-g1ucose. [^18^F] FDG is administered by intravenous injection and its concentration within the tissues is directly proportional to the glucose consumption rate; in cancer cells, an increase of glucose metabolism is usually observed [70]. [^18^F] FDG is transported inside the cell by glucose transporter proteins (GLUT) and then phosphorylated by Hexokinase enzyme to [^18^F] FDG-6-phosphate. Hexokinase activity and expression are related to tissue metabolism [70]. [^18^F] FDG-6-phosphate is not further metabolized and it can exit the cell only after being dephosphorylated by glucose-6-phosphatase. The products of dephosphorylation presumably leave cells by passive diffusion. The results on [^18^F] FDG kinetics suggest that the optimal bio-distribution of the radiopharmaceutical is generally achieved between 30 to 40 min after administration [70]. The physiological uptake of [^18^F] FDG in the brain is high with action potentials and postsynaptic effects being responsible for consuming much of the energy (47% and 34%, respectively), with the resting potential consuming a smaller amount (13%) [71]. Lastly, [^18^F] FDG might show variable uptake by inflammatory lesions [72]. Especially for low-grade gliomas, where a limited [^18^F] FDG consumption is expected, the aspects mentioned previously severely limits the tumor detection and evaluation [23]. Nevertheless, [^18^F] FDG PET/CT might reflect a reduced differentiation of the tumor (as detectable by an increased glucose consumption), thus providing prognostic information: in children with anaplastic astrocytomas, the degree of [^18^F] FDG uptake was positively correlated with the histopathologic tumor grade [24]. PET/CT with [18F] FDG imaging showed a prognostic value by metabolically estimating active tumor burden [25]. In a population of 40 children with a newly diagnosed diffuse intrinsic brain stem glioma, despite a poor survival, irrespective of the intensity of [^18^F] FDG, an uptake of [^18^F] FDG in the tumor superior to that of the gray matter suggested poorer survival rates [26]. An uptake of [^18^F] FDG in 50% or more of the tumor was related to poor progression-free survival (PFS) and overall survival (OS) [26]. As compared to MRI, the authors showed a significant relationship between [^18^F] FDG uptake and contrast enhancement [26]. In another report, PET/CT imaging with [^18^F] FDG showed promising results in the identification and stratification of high-risk low-grade gliomas. In 46 pediatric patients with ages that ranged from five months to 17 years affected by low-grade astrocytomas, an increased [^18^F] FDG uptake was related to higher malignancy. The authors concluded that [^18^F] FDG could be useful in the detection of progressive low-grade astrocytomas, and thus could be helpful in treatment management and planning [27]. In another study on 20 pediatric patients (age range 3–14 years) with brain-stem glioma, Kwon JW et al. concluded that the [^18^F] FDG PET uptake patterns are different in anaplastic astrocytoma and glioblastomas [28]. Figure 4 depicts a clinical case characterized by high [^18^F] FDG uptake in a temporal anaplastic astrocytoma.

Hexakis (2-methoxyisobutylisonitrile) technetium-99m ([^99m^Tc] MIBI) ([99mTc], half-life = 6.0067 h) is a cationic lipophilic agent [10]. Reconstruction is usually performed by adding an adequate activity of [^99m^Tc] O4^-^ to the kit and warming the mixture in boiling water according to the manufacturer’s recommendations. Although [^99m^Tc] MIBI prevalent use in clinical practice is as a myocardial perfusion imaging agent, MIBI was extensively applied, especially before the advent of PET technology, for the scintigraphic visualization of many tumors [73]. The mechanism by which MIBI is incorporated by malignant cells has only been partially elucidated, but several pieces of evidence demonstrated that MIBI incorporation in cells is strictly related to mitochondrial and plasma membrane potential [74]. It has to be highlighted that, MIBI can be transported out of the cells through an active mechanism after its internalization, against the cellular potential gradient, mediated by a 170-kDa plasma-membrane glycoprotein (Pgp). Pgp is also responsible for the resistance to several chemotherapic drugs, thus MIBI was used for the imaging of multidrug in several malignancies [74].

[^99m^Tc] Tetrofosmin (1,2- bis [bis (2-ethoxyethyl) phosphino]-ethane)is a lipophilic complex that has been introduced as an alternative imaging agent with respect to [^99m^Tc] MIBI for myocardial scintigraphy. From a technical point of view, the main advantage of tetrofosmin is its easy labeling without the need to heat the compound. Its mechanism of incorporation in malignant cells is similar to that described for [^99m^Tc] MIBI.

As regarding the application of [^99m^Tc] MIBI for the imaging of pediatric brain tumors, O’Tuama evaluated the clinical usefulness of SPECT with [^99m^Tc] MIBI in 19 children affected by intracranial malignancies in 1993, 16 of whom were assessed after treatment [12]. SPECT findings were correlated with the clinical status of the disease, being defined as “active” (when clinical examination showed a global worsening or CT/MRI depicted growing brain mass) or “inactive” (when radiological or clinical examination demonstrated stability or recent histology was negative for viable residual tumor). SPECT with [99mTc] MIBI was able to correctly disclose viable tumor tissue in PBT after therapy, even though two, not glial-lesions (i.e., medulloblastoma and dysgerminoma) resulted no-MIBI-avid. The main drawback of [^99m^Tc] MIBI resulted in its intense physiological uptake in the choroid plexus, which might hamper the detection of lesions located in the deep para-ventricular spaces. The premedication with potassium perchlorate was recommended to overcome these limitations and reduce the uptake in the choroid plexus.

In a study that was performed by Kirton and colleagues, the correlation between MIBI SPECT and MRI findings were analyzed in 20 pediatric subjects [13]. All of the patients were submitted to both MIBI and MRI before surgery, while six also underwent monitoring of the response to treatment over time. In the cited paper, most of the examined tumors were astrocytomas in which the authors found an excellent correlation between [^99m^Tc] MIBI uptake and MRI alterations. Of note, the grade of MIBI uptake was also dependent on the histology of the malignancy. It has to be pointed out that, among the examined tumors, false-negative reports (negative SPECT [^99m^Tc] MIBI vs positive MRI) were registered for the following lesions: optic glioma, medulloblastoma, desmoplastic neuroepithelial tumor, craniopharyngioma, spinal anaplastic astrocytoma, low-grade astrocytoma, and ependymoma. As regarding the role of [^99m^Tc] MIBI for monitoring patients after therapy, in a case with relapse of brain stem glioma SPECT was able to disclose recurrence earlier than MRI.

Barai and colleagues evaluated 21 patients with histologically proven lesions of the posterior cranial fossa as regards the use of SPECT with [^99m^Tc] Tetrofosmin for the imaging of PBT [14]. Among the examined subjects, twelve were children. All of the enrolled patients underwent SPECT with [^99m^Tc] tetrofosmin 8–36 months after radiotherapy. Of note, the prevalent histotype was represented by medulloblastoma. The SPECT findings were compared with follow-up and with the results of contextually performed contrast-enhanced CT: in the pediatric group, imaging with [^99m^Tc] Tetrofosmin was able to disclose only one of the seven cases with recurrent/residual viable tumor, thus showing very limited sensitivity. Therefore, the authors concluded that, although SPECT with [^99m^Tc] Tetrofosmin or MIBI might be considered for the evaluation of supratentorial lesions, its application for the lesions in the posterior fossa is not recommended. However, it has to be pointed out that none of the aforementioned researches with MIBI or [^99m^Tc] Tetrofosmin were performed by using hybrid SPECT/CT imaging. As concerns brain tumors in adults, the use of the correlation between morphological (i.e., CT) and functional (i.e., SPECT) data was found to be able to significantly increase the accuracy of visualization of intracranial lesions before and after radiation therapy [70]. Nevertheless, to the best of our knowledge, the hybrid SPECT/CT approach has not been specifically applied in pediatric neurooncology, most probably in relation to the diffusion of more effective PET-CT technology.

## 5. Imaging of Amino-Acid Metabolism and DNA Synthesis: [^18^F]-L-Dihydroxyphenylalanine ([^18^F] FDOPA), l-[Methyl-^11^C] Methionine ([^11^C]MET), O-(2-[^18^F]Fluoroethyl)-l-Tyrosine ([^18^F] FET), and 3′-Deoxy-3′-^18^F-Fluorothymidine ([^18^F] FLT)

In the last decade, several reports emphasize the possible role of amino acid tracers as l-[methyl-^11^C] Methionine ([^11^C] MET, half-life 20 mins.), [^18^F]-L-dihydroxyphenylalanine ([^18^F] FDOPA, half-life 108 mins.), and O-(2-[^18^F]Fluoroethyl)-L-tyrosine ([^18^F] FET, half-life 108 min) for the evaluation of brain tumors: this has been recently outlined in the latest clinical guidelines of the European Society of Nuclear Medicine [9]. [^18^F]-L-dihydroxyphenylalanine ([^18^F] FDOPA) is a large amino acid that is transported within the neurons. The enzyme aromatic amino acid decarboxylase [AAAD]) is responsible for the metabolism to [^18^F] FDOPA that enters catecholamine-storage vesicles [75]. [^18^F] FDOPA is able to cross the blood-brain barrier and is used in the brain as a precursor for dopamine neurotransmitters. For this reason, [^18^F] FDOPA has been used for the diagnosis of those neurodegenerative diseases that [75]. Another characteristic of [^18^F]-FDOPA is the increased uptake into cancer cells due to the overexpression of (predominantly L-type) AA transporters in several types of tumors. [^18^F] FDOPA may enter various pathways (e.g., peptide, protein, purine, pyrimidine, or hormone synthesis; act as methyl group donors, etc.) [76,77]. In several types of tumors, the malignant transformation increases the use and metabolism of amino acids as a source of energy mostly used for protein synthesis and cell division, thus leading to an overexpression of transporter systems [78]. [^11^C] MET and [^18^F] FET are two radiopharmaceuticals that can easily cross the blood-brain barrier and they are taken up in the brain by L-type amino acid transporter system and Na^+^-dependent system B0 and are then mainly incorporated into proteins, but also into lipid, RNA, and DNA. The systems that are responsible for [^11^C] MET and [^18^F] FET uptake and metabolism represent the target of imaging with this radiopharmaceutical [79,80]. Moreover, the uptake of [^18^F]-FDOPA also reflects the Aromatic L-amino acid decarboxylase activity [75].

The radiopharmaceutical 3′-Deoxy-3′-^18^F-fluorothymidine ([^18^F] FLT, half-life 108 min) (an analog of Thymidine), is phosphorylated by a cytosolic enzyme that is high in proliferating cells [thymidine kinase-1, (TK-1)]. The uptake and accumulation of FLT can be used as an index of cellular proliferation. The greater expression of this enzyme is during the S-phase of the cell cycle [81]. Differently from thymidine, [^18^F] FLT monophosphate is not incorporated into DNA and it is impermeable to the cell membrane and similarly to [^18^F] FDG is not further metabolized and trapped inside the cells [75].

Molecular imaging of amino-acid metabolism and DNA synthesis provides a unique opportunity for the better characterization of the tumor at a molecular level. Differently from the radiopharmaceuticals mentioned above (in particular [^18^F] FDOPA and [^18^F] FDG ), [^18^F] FLT shows reduced uptake in normal brain cortex, thus allowing for better detection of neoplastic tissues [9]. To the best of our knowledge, few studies have evaluated the usefulness of radiopharmaceuticals that are designed for the imaging of amino acid metabolism in PBT, but the preliminary results on the utility and the limited data available (reported below) suggest that radiolabeled amino acids have similar diagnostic utility in pediatric and adult neuro-oncology [9,82,83].

^11^C MET is one of the most widely used radiopharmaceuticals for the evaluation of malignant brain tumors. O’Tuama et al. [29] reported the first experience with [^11^C] MET PET in childhood brain tumors: their study demonstrated abnormal accumulation of MET in 85% children (age range 1.8–15.8 years old) with PBT. The mean ratio of [^11^C] MET uptake in tumor vs. normal brain was 1.5 +/− 0.57; range: 1.13–2.98) [29]. Increased tracer uptake was observed in different tumors phenotypes as ependymomas, medulloblastoma, and astrocytomas with the lower uptake being detectable in low-grade tumors [29]. Utriainen et al. have shown that [^11^C] MET uptake in pediatric brain tumors is positively associated with tumor grade [30]. In particular, the apoptotic index correlated significantly with [^11^C] MET uptake, while no significant associations were observed with Ki-67 expression [30]. Pirotte et al. [31] demonstrated that PET imaging with [^18^F] FDG and [^11^C] MET was helpful in the surgical management of 126 children with PBT, surgical, and post-operative steps. In the cited report of Pirotte et al. [31], incidental lesions were clearly classified with PET by detecting tumor/evolving tissue. During the diagnostic course of the disease, [^11^C] MET PET guided the stereotactic biopsies in gliomas facilitating the number of trajectories in biopsies. The major advantage of PET guided biopsy was evident in those procedures that involved the brainstem or the pineal region. As for surgical planning, [^11^C] MET PET allowed for better identification of tumor infiltration along the functional cortex than MRI optimizing the resection of the tumor (focused also on more active areas) and significantly increasing the volume of the resected tissue that is a key factor for survival in PBT [31]. Lastly, [^11^C] MET improved the detection of tumor residues in the operative cavity at the early postoperative stage, thus facilitating the decision of early second-look surgery for optimizing the radical resection [31].

[^11^C] MET PET detected recurrent tumors in 90% of the cases and allowed for the delineation of those regions at increased risk for recurrence thus facilitating the target definition for radiotherapy in another report of Lucas Jr. et al. [32] on a population of more than 30 children with high-grade gliomas.

**Figure 5:**
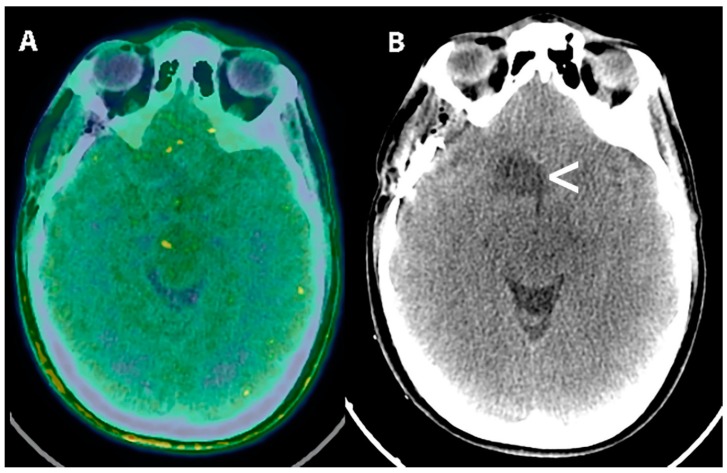
[^18^F] FDOPA PET (**A**) and T2 FLAIR MRI (**B**) in a 14-year-old female patient with the suspect of primary brain tumor. T2 FLAIR image showed an area of mild hyperintensity in the left temporal lobe(**B**,<). [18F] FDOPA PET/CT scan demonstrated increased uptake of the radiopharmaceutical in the lesion with a Standardized uptake value equal to 2.2 vs. 1.1 of the surrounding brain tissue (**A**,<). The patient was surgically treated and the subsequent diagnosis of low-grade glioma (WHO grade II).

**Figure 6:**
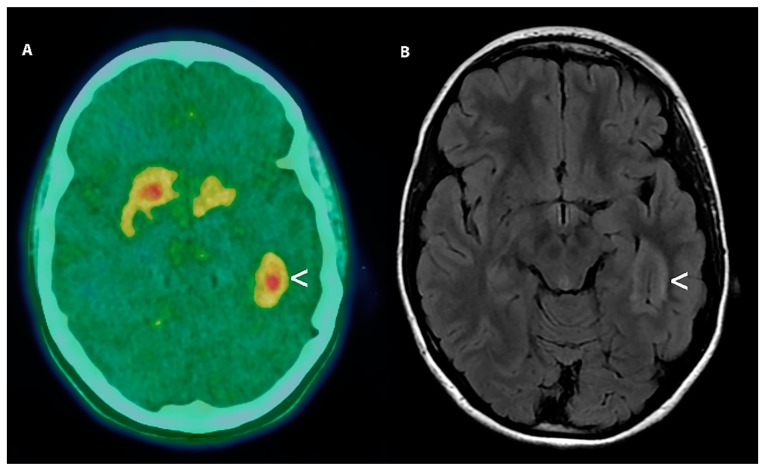
[^18^F] FDOPA PET (**A**) performed on September 2013 of a 16 years old male patient surgically treated for a Pilocytic Astrocytoma showing no area of focal increased uptake of the radiopharmaceutical (Standardized uptake value was equal to 0.9 vs. 0.8 of the surrounding brain tissue) (**A**,<). In (**B**) we report the axial CT scan showing the extent of surgery. Routine follows up examinations in February 2019 was negative for cancer recurrence.

[^18^F] FDOPA has demonstrated high potential in defining tumor grade and outcome in PBT. Figure 5 and Figure 6 provide examples of [^18^F] FDOPA PETscan in a patient with PBT.

Morana et al. performed a comparative analysis on diagnostic and prognostic value of [^18^F] FDOPA PET and MR spectroscopy in PBT in 27 pediatric patients with supratentorial infiltrative brain lesions on conventional MRI. The authors found that [^18^F] FDOPA uptake was significantly related to PFS and OS [33]. Spectroscopy did not show a significant association with prognosis when also considering the Choline/N-Acetyl-Aspartate ratio (Cho/NAA ratio) [33]. The wide distribution of scanners in the facilities, together with the relative low cost and radiation exposure, makes MRI one of the most used diagnostic tools in differentiating brain gliomas from non-neoplastic lesions, while [^18^F] FDOPA perform better in discriminating between low-grade and high-grade gliomas [33]. In another report, Morana et al. [34] showed that [^18^F] FDOPA uptake is related to the prognosis. In this study on 26 patients with PBT, the patients were investigated with MRI derived diffusion-weighted imaging (DWI) and arterial spin labeling (ASL) perfusion imaging and [^18^F] FDOPA PET within two weeks. [^18^F] FDOPA uptake resulted in the best independent predictors of survival on multivariate analysis [34]. When combining DWI, ASL, and [^18^F] FDOPA PET data, the authors obtained the best predictor for tumor progression, thus suggesting that the combination of different imaging modalities represents an added value for the evaluation of malignancies, as also proposed for other diseases [34,84]. This aspect was further addressed in another report of Morana G et al. [35]. The accuracy, sensitivity, specificity, positive predictive value (PPV), and negative predictive value (NPV) were 93%, 89%, 100%, 100%, and 82% for [^18^F] FDOPA PET/MRI, and 75%, 74%, 78%, 88%, and 58% for [^18^F] FDOPA PET/CT with a major advance of the combination of PET with MRI data for the evaluation of striatal involvement in PBT due to the physiological uptake of [18F] FDOPA in basal ganglia (see above) [35].

When performing diagnostic investigations, inpatients with PBT after treatment, one should consider that antiangiogenic therapy can decrease contrast enhancement soon after the administration of the drug (as early as 24 to 48 h) [36]; Morana et al., in a case report, noticed that in high-grade PBT the treatment with bevacizumab and temozolomide was associated to a significant reduction of the enhancement at MRI [37], while [^18^F] FDOPA at the same time point showed a high uptake of the radiopharmaceutical in the non-enhancing component of the tumor [37]. A 16-week MRI revealed significant progressive disease, which indicated that contrast-enhanced MRI is not an adequate early predictor of response to antiangiogenic therapy and [^18^F] FDOPA PET may have a potential promising role in distinguishing tumor pseudoresponse and progression [37].

Gauvain et al. [38] demonstrated the safety and feasibility of [^18^F] FDOPA PET/MRI in monitoring the response to bevacizumab early during the course of chemotherapy in relapsed PBT. The authors observed a range of responses to four weeks of bevacizumab, as assessed by changes in the uptake of [^18^F] FDOPA suggesting that this imaging modality may predict response at three months after the beginning of chemotherapy. When considering the small population that was examined in this study, future studies on a larger number of subjects are necessary and no definitive conclusions can be made regarding the role of this technique in assessing response to therapy [38].

[^18^F] FET showed promising results in biopsy guidance and treatment planning of cerebral tumors, especially when compared to [^18^F] FDG [39,40]. [^18^F] FET imaging showed good agreement with tissue sampling for differentiating therapy-associated changes from tumor progression and for identifying metabolic “hot spots” in newly diagnosed lesions and in surgical planning [42,43,44]. [^18^F] FET imaging was useful for tumors located in the brainstem and spinal cord [45] and in incidental brain lesions; the association between limited growth pattern on MRI and low [^18^F]-FET uptake are associated with a more benign course [46]. It has been recently noticed that [^18^F] FET uptake is significantly increased in IDH1/2 mutant–1p/19q non-codel gliomas. The availability of novel and standardized tools for image analysis could be used for in vivo molecular marker analysis, while considering the major importance in the new World Health Organization (WHO) classification [47]. For a similar purpose, other Authors investigated the role of dynamic [^18^F] FET PET in H3-G34-mutant gliomas. These authors found an association with an extensive and diffuse tracer uptake pattern that was similar to that of aggressive high-grade gliomas, with disagreement with MRI in most of the cases [48].

As for [^18^F] FLT, to the best of our knowledge, the reports on PBT are very limited. Grant F et al. recently published the preliminary reports of a study performed in 22 pediatric patients (age 6–21 years) in which MRI suggested a newly diagnosed or recurrent brain tumor [85]. Tumor uptake of [18F] FLT, as assessed by SUVmax ranged from 0.11 (oligoastrocytoma, with uptake similar to background brain) to 4.00 (pilocytic astrocytoma). The Ki-67 labeling index (fraction of cells staining with MIB antibody) ranged between 3% and 66%, in keeping with a wide range of tumor types and grades. A pilocytic astrocytoma was the most common tumor type (*N* = 4). In the 17 participants with new or recurrent brain tumors, tumor uptake of FLT (SUVmax) significantly correlated with Ki-67 labeling index (Pearson *r* = 0.74, *p* < 0.001). In the nine participants with possible tumor recurrence, SUVmax ranged from 1.54 to 4.00, and they were all demonstrated to have tumor recurrence [85]. These findings suggest that [^18^F] FLT is able to predict the degree of cell proliferation in PBT and they can help in the detection of tumor recurrence.

## 6. Ongoing Clinical Studies and Future Perspectives

In the last years, growing scientific interest has been focused on the role of specific membrane antigens expressed by malignant cells, as potential targets for molecular imaging and therapy. In this regard, a promising role seems to be played by the monoclonal antibody bevacizumab labeled with the radionuclide zirconium-89 ([^89^Zr], half-life: 78.4 h). Bevacizumab represents an IgG1 antibody directed against vascular endothelial growth factor A (VEGF-A) and it is widely used for the therapy of different types of cancers, such as colorectal cancer, metastatic non-squamous non–small cell lung cancer (NSCLC), and recurrent glioblastoma [86,87]. In this clinical setting, PET with [^89^Zr] bevacizumab has been applied for the in vivo assessment of patients’ individual suitability for treatment with bevacizumab [88].

Recent molecular studies have revealed the high expression of VEGF in diffuse intrinsic pontine glioma (DIPG), one of the most aggressive PBT [89]. The evidence of high VEGF expression in DIPG triggered the start of several clinical trials that aimed at assessing the potential therapeutic value of bevacizumab in this pediatric neurooncological setting (i.e., https://clinicaltrials.gov/ct2/show/NCT01182350). As concerns the role of molecular imaging in this field, Veldhuijzen van Zanten et al. evaluated the role of PET with [^89^Zr] bevacizumab for determining whether or not patients with DIPG may benefit from treatment with the anti-VEGF monoclonal antibody [50]. The authors performed a PET scan with [^89^Zr] bevacizumab in a pediatric patient that was affected by DIPG and then carried out a correlation analysis between PET semiquantitative parameters, tumor histology, and vascularization at post more examination. [^89^Zr] bevacizumab uptake was found to closely correlate with the vascular proliferation and the histochemical heterogeneity within the tumoral lesion, thus suggesting the potential usefulness of this molecular imaging probe in determining the suitability for antiangiogenetic therapy in DIPG patients.

Another scientific topic that is gaining increasing attention in oncology is represented by the immunological environment in which cancer develops and grows. In particular, the interaction between the host’s adaptive response and the tumor has been demonstrated to have a crucial role in many clinical scenarios, since malignancy promotion and progression are strictly related to immunosuppression. The recently introduced monoclonal antibodies that are directed against specific immune checkpoint inhibitors, such as programmed death-1 (PD-1) and cytotoxic T-lymphocyte antigen-4 (CTLA-4), are providing promising results in different malignancies [90]. However, the role of immunotherapy in PBT has not yet been deeply investigated. Preliminary data suggested that therapy with the immune-modulating antibody MDV9300 might be effective in the treatment of children affected by high-grade gliomas [91]. In a recently published review by Wierstra et al., different tracers for the PET/SPECT imaging of patients submitted to immunotherapy were evaluated [92]. In particular, monoclonal antibodies targeting PD-1, such as pembrolizumab, have been labeled with the radionuclide copper-64 ([^64^Cu], half life = 12.7 h) or with [^89^Zr]. These radio compounds provided preliminary satisfying results for detecting PD-1 expression in malignant lesions, thus representing potentially useful tools for identifying patients who are most likely to benefit from immunotherapy. However, further studies with larger series are needed to better define the implications of immunotherapy and its molecular imaging counterpart in the pediatric neuro-oncological field.

## 7. Conclusions

Preliminary results of the applications of MI in PBT are encouraging. SPECT and PET radiopharmaceuticals have the ability of molecular properties and pathophysiological changes in the tumor, thus allowing for an accurate diagnosis bot at staging and after treatment. MI will play a crucial role in the management of PBT, given the present paradigm shift in medicine from reactive to proactive approaches. The advantages are mostly related to a tailored treatment by carrying radiolabeled compounds to a target-specified site. For this reason, MI should be considered to be an important imaging modality in addition to MRI for improving the accuracy in various aspects of PBT management.

## Figures and Tables

**Table 1:** Summary of the most relevant manuscripts on the use of single-photon emission computed tomography (SPECT) and positron emission tomography (PET) probes in pediatric brain tumors. [^99m^Tc]-Methoxyisobutylisonitrile ([^99m^Tc] MIBI); [^11^C] Choline ([^11^C] CH); [18F]fluoroethylcholine ([18F] FEC); [111In] -diethylenetriaminepentaacetic acid-d-phenylalanine-octreotide ([111In] pentetreotide); O-(2-[18F]Fluoroethyl)-l-tyrosine ([18F] FET); [18F] Fluorodeoxyglucose ([18F] FDG); [18F]-L-dihydroxyphenylalanine ([18F] FDOPA); l-[methyl-11C] Methionine ([^11^C]MET); [18F]-L-dihydroxyphenylalanine ([18F] FDOPA).

Authors	Year	Radiopharmaceutical	Type of Study	Setting	Patients	Comment	Type of Population
O’ Tuama et al. [12]	1993	[^99m^Tc] MIBI	Case series	Pre-operative imaging Restaging post-therapy	*N* = 19	SPECT with MIBI was able to detect primary brain tumors, especially gliomas, but lesions in the para-ventricular spaces are of difficult visualization.	Pediatric
Kirton et al. [13]	2002	([^99m^Tc] MIBI	Case series	Pre-operative imaging Monitoring after therapy	*N* = 20	SPECT with MIBI correlates with MRI in astrocytomas, but present reduced sensitivity in disclosing some histotypes such as medulloblastoma and optic glioma. MIBI was able to disclose recurrence earlier than MRI.	Pediatric
Barai et al. [14]	2003	[^99m^Tc]-Tetrofosmin	Case series	Restaging post radiotherapy	*N* = 12	SPECT with tetrofosmin was not accurate for the detection of recurrent tumors in the posterior cranial fossa.	Mixed
Ohtani et al. [15]	2001	[^11^C] CH	Prospective, single-center	Pre-operative imaging	*N* = 3	PET-CT with ^11^C-choline performed better than ^18^F-FDG for the detection of brain lesions but failed in discriminating low-grade gliomas and non-neoplastic lesions.	Mixed
Fraioli et al. [16]	2015	[^18^F] FEC	Prospective, single-center	Pre-operative imaging Restaging post-therapy	*N* = 12	PET-MRI with a hybrid scanner may represent a useful diagnostic tool in pediatric astrocytomas. An inverse correlation trend was found between SUV_max_ and ADC.	Pediatric
Tsouana et al. [17]	2015	[^18^F] FEC	Case series	Pre-operative imaging Restaging post-therapy	*N* = 4	PET-MRI with ^18^F-choline was able to correctly characterize intracranial non-germinomatous germ cell tumors and monitor the response to chemotherapy.	Adolescent
Muller et al. [18]	1998	[^111^In] pentetreotide	Case series	Pre-operative imaging Restaging post-therapy	*N* = 16	Somatostatin receptor imaging with ^111^In-pentetreotide identified medulloblastoma before surgery and residual viable tissue after therapy.	Pediatric
Frühwald et al. [19]	2004	[^111^In] pentetreotide	Case series	Restaging post-therapy	*N* = 13	Somatostatin receptor imaging with ^111^In-pentetreotide was able to detect residual disease or relapse in selected pediatric brain tumors.	Pediatric
Abongwa et al. [20]	2017	[^68^Ga]DOTATOC	Prospective Clinical Trial	Safety Study	*N* = 2	Safety and accuracy of ^68^Ga-DOTATOC PET/CT in children and young adults with solid tumor	Mixed
Arunraj et al. [21]	2018	[^68^Ga]DOTANOC	Case report	Restaging post therapy	*N* = 1	^68^Ga-DOTANOC PET is able to detect medulloblastoma recurrence.	Adolescent
Menda et al. [22]	2010	[^90^Y]DOTANOC	Phase I study	Safety and efficacy of PRRT	*N* = 17	^90^Y-DOTANOC presented a favorable safety profile and an overall response rate of 76% in refractory children tumors overexpressing somatostatin receptors.	Mixed
Dunkl et al. [6]	2015	[^18^F] FET	Case series	Pre-operative imaging Restaging post-therapy	*N* = 49	PET with FET was helpful in decision making in PBT.	Pediatric
Misch et al. [7]	2015	[^18^F] FET	Case series	Pre-operative imaging PET guided surgical biopsy and resection	*N* = 26	Biopsy guided by PET with FET increased the accuracy of histological diagnosis with decent specificity and high sensitivity	Pediatric
Law et al. [9]	2019	[^18^F] FET; ([^11^C]MET); ([^18^F] FDOPA)	Practice guidelines	Pre-operative imaging Monitoring after therapy Restaging post-therapy		Guidelines aimed to assist nuclear medicine practitioners in recommending, performing, interpreting and reporting the results of brain PET with MET, FET, and FDOPA.	-
Kim et al. [23]	2010	[^18^F] FDG; [^11^C]MET	Review article	Pre-operative imaging		The usefulness of PET and PET/CT in the evaluation of pediatric pediatric brain tumors.	-
Uslu et al. [24]	2015	[^18^F] FDG	Review article	Pre-operative imaging		The usefulness of FDG PET/CT in the evaluation of pediatric malignancies and the role of PET/MR in the reduction of radiation exposure.	-
Williams et al. [25]	2008	[^18^F] FDG	Case series	Pre-operative imaging Monitoring after therapy	*N* = 12	3D PET for the estimation of metabolically active tumor burden; possible prognostic value after tumor grade is determined	Pediatric
Zukotynski et al. [26]	2011	[^18^F] FDG	Case series	Pre-operative imaging Monitoring after therapy	*N* = 40	Prognostic value of FDG PET in PBT.	Pediatric
Kruer et al. [27]	2009	[^18^F] FDG	Case series	Pre-operative imaging Monitoring after therapy	*N* = 46	The role of PET in high-risk Low-grade astrocytomas.	Pediatric
Kwon et al. [28]	2006	[^18^F] FDG	Case series	Pre-operative imaging Monitoring after therapy	*N* = 20	The role of FDG-PET in differentiating between anaplastic astrocytoma and glioblastomas among high-grade tumors	Pediatric
O’ Tuama et al. [29]	1990	[^11^C]MET	Case series	Pre-operative imaging Restaging post-therapy	*N* = 13	The role of PET with MET in PBT: differential diagnosis between tumor recurrence and cerebral radiation injury.	Pediatric
Utriainen et al. [30]	2002	[^18^F] FDG; [^11^C]MET	Case series	Pre-operative imaging Restaging post-therapy	*N* = 27	Association between FDG and MET uptake and malignancy grade in PBT.	Pediatric
Pirotte et al. [31]	2007	[^18^F] FDG; [^11^C]MET	Case series	Pre-operative imaging Restaging post-therapy	*N* = 126	The role of PET imaging in the surgical management of PBT at the diagnostic, surgical, and post-operative steps	Pediatric
Lucas at al. [32]	2017	[^11^C]MET	Case series	Pre-operative imaging Restaging post-therapy	*N* = 31	The role of MET PET in PBT at increased risk for recurrence	Pediatric
Morana et al. [33]	2015	[^18^F] FDOPA	Retrospective comparative study	Pre-operative imaging Monitoring after therapy	*N* = 27	The role of FDOPA in discriminating low-grade from high-grade gliomas	Pediatric
Morana et al. [34]	2017	[^18^F] FDOPA	Retrospective study	Pre-operative imaging Monitoring after therapy	*N* = 26	Combination of MRI and FDOPA PET show the highest predictive power for prognosticating PBT progression	Pediatric
Morana et al. [35]	2016	[^18^F] FDOPA	Retrospective study	Pre-operative imaging Monitoring after therapy	*N* = 28	The technical paper aimed to investigate the physiological striatal FDOPA uptake in the evaluation of basal ganglia involvement of PBT in PET/TC.	Pediatric
Hutterer et al. [36]	2015	[^18^F] FDG; [^18^F] FET; [^11^C]MET; [^18^F] FDOPA	Review article	Pre-operative imaging Monitoring after therapy		Paper aimed to investigate multimodal imaging that combines standard and advanced MRI with amino acid PET imaging to detect drug susceptibility or resistance of PBT	
Morana et al. [37]	2013	[^18^F] FDOPA	Case report	Pre-operative imaging Monitoring after therapy	*N* = 1	The role of FDOPA PET in distinguishing tumor pseudoresponse and progression.	Pediatric
Gauvain et al. [38]	2018	[^18^F] FDOPA	Case series	Pre-operative imaging Monitoring after therapy	*N* = 6	The role of FDOPA PET/MRI in the early prediction of therapy response at 3 months	Pediatric
Pauleit et al. [39]	2008	[^18^F] FDG; [^18^F] FET	Comparative study	Pre-operative imaging Monitoring after therapy	*N* = 52	FET PET is superior to FDG for biopsy guidance and treatment planning of cerebral gliomas.	Adult
Plotkin et al. [40]	2010	[^18^F] FDG; [^18^F] FET	Comparative study	Pre-operative imaging Monitoring after therapy	*N* = 15	FET PET is superior to FDG for biopsy planning in non-contrast-enhancing PBT.	Adult
Pauleit et al. [41]	2005	[^18^F] FET	Case series	Pre-operative imaging Monitoring after therapy	*N* =31	The combined use of MRI and FET PET in patients with cerebral gliomas improves the identification of cancer recurrence	Adult
Floeth et al. [42]	2005	[^18^F] FET	Case series	Pre-operative imaging Monitoring after therapy	*N* =50	The role of FET-PET and MR spectroscopy in patients with intracerebral lesions: efficacy of targeted biopsies.	Mixed
Pöpperl et al. [43]	2014	[^18^F] FET	Case series	Pre-operative imaging Monitoring after therapy	*N* =53	The role of FET PET reliably in distinguishing between post-therapeutic benign lesions and tumor recurrence after treatment in low and high-grade gliomas.	Mixed
Stockhammer et al. [44]	2009	[^18^F] FET	Case series	Pre-operative imaging	*N* =13	FET-PET provides useful information for planning glioma resection.	Adult
Tscherpel at al. [45]	2017	[^18^F] FET	Case series	Pre-operative imaging	*N* = 36	Added-value of FET PET in the brainstem and spinal cord glioma, particularly when MRI is equivocal	Mixed
Floeth at al. [46]	2008	[^18^F] FET	Prospective clinical trial	Pre-operative imaging Monitoring after therapy	*N* = 21	The prognostic role of FET uptake for the development of a high-grade glioma	Mixed
Suchorska et al. [47]	2018	[^18^F] FET	Case series	Pre-operative imaging Monitoring after therapy	*N* = 300	Differences in FET uptake patterns in subgroups of IDH1/2 mutant-1p/19q non-codel gliomas	Mixed
Vettermann at al. [48]	2018	[^18^F] FET	Case series	Pre-operative imaging Monitoring after therapy	*N* = 8	Paper on FET PET uptake patterns in high-grade glioma with H3-G34-mutation.	Mixed
Utriainen et al. [49]	2003	[^11^C] CH	Case series	Pre-operative imaging Monitoring after therapy	*N* = 12	^11^C-choline PET uptake patterns differ according to the proliferative activity of tumors	Adult
Fraioli at al. [16]	2015	[^18^F] FEC	Case series	Pre-operative imaging Monitoring after therapy	*N* = 12	Coupled imaging of ^11^C-choline and MRI in children with astrocytic tumors	Pediatric
Veldhuijzen van Zanten et al. [50]	2018	[^89^Zr] Bevacizumab	Case report	Pre-operative imaging	*N* = 1	Correlation between tracer uptake and tumor’s histochemistry at post mortem examination	Pediatric

**Table 2:** Summary of the different molecular probes used for pediatric brain tumor imaging, with their respective advantages and drawbacks.

Molecular Imaging Probe	Advantages	Drawbacks
[^11^C] choline	High uptake in gliomas.	[^11^C] short half-life
		High uptake in choroid plexus
[^18^F] FEC [^18^F] FCH	High uptake in brain tumors. The adequately long half-life of the [18F] adionuclide	High uptake in choroid plexus
[^68^Ga]-DOTATOC [^68^Ga]-DOTANOC [^68^Ga]-DOTATE	In vivo detection of receptorial status. Potential therapeutic implications if labeled with [177Lu] or [90Y]	Need for on-site generator Sensitivity limited to brain tumors expressing somatostatin receptors (i.e., medulloblastoma, pineoblastoma, etc.)
[^99^mTc] MIBI [^99^mTc] Tetrofosmin	Widespread availability in the majority of the nuclear medicine centers	Poor spatial resolution. High uptake in the choroid plexus. Poor diagnostic accuracy for tumors of the posterior cranial fossa.
[^18^F] FDG	High uptake of [^18^F] FDG may reflect a poor differentiation of the tumor.	High Physiological uptake of [^18^F] FDG in the brain limits the tumor evaluation and detection. Moreover, [^18^F] FDG may show variable uptake by inflammatory lesions.
[18F] FDOPA [^18^F] FLT [^11^C] MET	Amino-acid tracers show a reduced uptake in the normal brain cortex, thus providing a better characterization of the tumor. Amino-acid uptake in pediatric brain tumors is correlated with tumor grade. High potential in defining the outcome in pediatric brain tumors.	Short Half-life of [^11^C] MET (20 min.); physiological uptake of [^18^F] FDOPA in basal ganglia may limit the evaluation of striatal involvement in brain tumors. Reports on pediatric brain tumors are limited.
[^18^F] FLT	Tumor uptake of [^18^F] FLT correlated significantly with the Ki-67 labeling index.	Reports on pediatric brain tumors are very limited.
